# Iran’s Experience in Establishing Convalescent Care Facilities for Patients With COVID-19

**DOI:** 10.1017/dmp.2020.209

**Published:** 2020-06-24

**Authors:** Ali Maher, Mohammadkarim Bahadori, Ramin Ravangard, Rouhollah Zaboli

**Affiliations:** Department of Health Policy, Economics and Management, School of Management and Medical Education, Shahid Beheshti University of Medical Sciences, Tehran, Iran; Health Management Research Center, Baqiyatallah University of Medical Sciences, Tehran, Iran; Health Human Resources Research Center, School of Management and Information Sciences, Shiraz University of Medical Sciences, Shiraz, Iran

**Keywords:** Covid-19, convalescent care, facilities, Iran

Policy-makers, health care providers, and the public are interested in getting services in the right place because providing services in the wrong place will have negative consequences for access, cost, and quality. The use of alternative care levels is one of the indicators of performance management and accountability to patients who do not require acute and long-term care^1^; although, there is yet no clear definition of such services in the world, and this is unclear among experts. There is substantial confusion about and overlap between health and social care services labeled as “subacute,” “intermediate care” and those labeled as “reablement.” In this article, we have used “convalescent.”^2,3^ The convalescent care facilities model is defined as a transitional form of care provided after a hospital stay but before going home. Convalescent care provides a home-like environment during post-surgery recovery, injury recovery, and can even be used as a transitional form of care following stroke or a lengthy illness. Previously, such services were mainly used for diseases, such as strokes, as well as accidents that the patient required convalescent services, and their effectiveness have been proven.^4^ The rapid outbreak of coronavirus disease (COVID-19) in Iran paved the way for the establishment of convalescent centers because one of the issues related to the break of the coronavirus transmission chain is that patients should not return home immediately after recovery and discharge from the hospital and instead they need intermediate places to recover completely from COVID 19 and reduce any chance of transmitting the disease. The health care system in Iran consists of 4 levels: the first level, which includes health houses and health posts; the second level, which consists of comprehensive health centers and clinics; the third level, which includes public hospitals; and the fourth level, which consists of specialty and subspecialty hospitals.^5^ In epidemics, another level of services is designed and operated, the convalescent centers, which is placed between the second and third levels of the service delivery system. Convalescent centers are an integral part of the service delivery system in Iran, which do not require advanced hospital facilities, and their responsibility is to admit suspected patients infected with disease from an epidemic or to admit patients who do not need specialized hospital services.

Due to the increasing number of patients with COVID-19 in Iran who are discharged from the hospitals to continue their additional and supplementary treatment and care at home but do not have adequate isolation space or a way to take care of themselves, establishing a temporary place such as convalescent centers for these patients and providing necessary services and training for them is an appropriate strategy. In Iran, convalescent centers were set up by military organizations, municipalities, non-governmental organizations, and the public themselves, and almost no government facilities were used to establish them. However, addressing the technical issues related to setting up, equipping, and monitoring the centers was the responsibility of medical universities. The physical space in these centers for hospital beds per bed is at least 5 meters. The health care team for every 50 beds includes 1 nurse, 2 practical nurses, 1 service personnel, and 1 guard; and the required equipment includes an emergency trolley and Cardiopulmonary and Cerebral Resuscitation equipment. At least 14 days after the onset of early symptoms, patients should be isolated in the convalescent centers.

In one of the military health care organizations, a 1000-bed convalescent center was established within 48 hours in the organization’s multi-story car park. Nurses and nursing students worked in shifts and in on-call form to organize the health care teams of this convalescent center. The Islamic Republic of Iran Army established a 2000-bed convalescent center in less than 24 hours at the Tehran International Exhibition Center. Moreover, the Iran Mall, one of the biggest shopping malls in the world, established a convalescent center with 3000 beds in 5 working days, so that in the first stage, 50 intensive care unit beds and 150 beds equipped with oxygen therapy equipment were used. This center was equipped with the latest and most up-to-date medical facilities and became a care center for COVID-19 patients. The establishment of these convalescent centers has gradually expanded to other cities in Iran.

Convalescent service providers should comply with professional standards for providing services to the patients. Due to the high risk of being infected with COVID-19 in the convalescent centers, it is recommended to observe the minimum social distance (6 feet) requirement in the arrangement of beds. The separation of clean and infected spaces and design of safe traffic routes are essential. Removing face-to-face contact with family members and prohibiting group activities increase the recovery time for socially isolated patients. One of the topics that should be considered in convalescence is appropriate leveling and space creation, which includes the spaces for COVID-19-suspected patients, the spaces for patients in good condition, and the spaces for people who have had physical contact with the patients. These spaces have not been considered in some convalescent centers, which are not based on epidemiological models of the needs assessment. The double stress of fear of re-infection leads to behavioral problems. Delirium caused by hypoxia, one of the important clinical features of COVID-19, can complicate the dementia. Therefore, in the convalescent centers, a protocol on how to provide mental health services should be developed.^3^ The convalescent center is a good opportunity for the cooperation of volunteer forces in the epidemic. The executive plan for the use of volunteers in the convalescent centers can compensate for the care workers, and the expert forces and staff in the hospitals can focus on providing services to the more acute patients. The health care system and the National Corona Committee should use tools to identify capacity to increase the standard convalescent beds and to securely transfer patient information. The use of Skype and other applications for secure virtual calls should be increased to ensure that the physician’s or health care advice is followed. Convalescent care audits, including scrutinizing the daily infection controls, nursing services, and the transparent reporting process of personal care plans for patients in the convalescent center, mental health measures, and finally additional activities such as providing balanced and delicious meals and physiotherapy services are all key indicators in the operation of a convalescent center during the COVID-19 epidemic.
